# Reforming a Reformer

**Published:** 1877-05

**Authors:** 


### *REFORMING A REFORMER.*

We have received a copy of a magazine denominated *The Health Reformer*, published monthly at Battle Creek, Michigan. It is the April issue, and we find in it a good deal of useful and entertaining matter. It discountenances the use of alcohol, tobacco and other poison, and advocates the employment of such potent remedial and supporting agencies as water, air, sunlight and exercise. In all this it is sensible, and deserves our warm commendation.

But a reformation is sadly demanded in the method of this Reformer, and to this end we will devote a little of our space. It copies from one of the editorials in our issue for October, 1876, without giving us credit, which is not quite courteous, to say the least. In the article quoted from, we mentioned the circumstance that the Daily *Witness* of this city had declined to publish the advertisement of the Health Food Co., because of the injunction embodied in said advertisement to "Avoid Graham" meal and other crude grains. The object of this "Reformer," in thus using our matter, is to say unkind things concerning HALL'S JOURNAL OF HEALTH and the Health Food Co., an object which it manages to accomplish by a well-nigh miraculous evasion of truth.

It speaks of us as "a journal evidently managed in the interest of" the Health Food Co., a statement utterly baseless. We have been the friend of this organization from its inception; have appreciated the earnest and self-sacrificing labors of its honored founder; have been made the confidant of the results of his many experiments upon man and animals, as well as of his diligent microscopical and chemical researches, and have not hesitated to say that the Health Food Company—which enjoys the benefit of his skill—is by far the best authority on subjects of alimentation to be found on either continent. This was the conviction of the late Dr. Hall, who took great interest in their labors and investigations, as our readers are aware. This feeling is shared by all the leading hygienists of our acquaintance, including very many who long followed the theories of Sylvester Graham with all their crudities, and who now rejoice in the better way. It is, as we know, the belief of hundreds of physicians of all schools, who are in the habit of seeking from this society that knowledge of food substances which is not as yet embodied in literature, or in the teachings of medical schools, and which is so essential to the successful treatment of nearly all diseased conditions.

This Battle Creek *Reformer* calls us conceited, and declares our statements false because we denominated Dr. Graham a "false prophet of hygiene," and asserted that bran bread and kindred trash are calculated to do serious harm. He tries to prove that the use of these husky foods has restored many sick persons to health, but he does not succeed.

As to our characterization of Sylvester Graham, we believe we do him equal and exact justice when we denominate him an able humbug. We think it probable that we know more about him than the Michigan doctor. We know that after publicly inveighing against tea, he drank seven cups of the strongest infusion at one sitting, and instead of eating the branny bread which he urged on others, he selected the whitest and finest for his own palate. If he was not a false prophet, and a falsehood, clear through, we never saw one. We could truthfully say some very hard things of Mr. Graham personally, but we haven't time.

As to his teachings we are positive that we echo the sentiment of the best-informed physiological chemists, when we declare that his food-philosophy was a sad mixture of truth and error, and we know it has been productive of vast injury to the American people.

As to the claim of this later "Reformer" that bran cures sick folks, we leave it, with the simple remark that the same thing is claimed for Dr. Brandreth's and Dr. Everybody's pills; that bran and Brandreth are both cathartic agents; that cathartics have no food value; that when people tell us they eat laxative bran or laxative pills habitually, and feel all the better for it, we naturally feel a little incredulous. While we do not deny that cathartics are sometimes useful, we are convinced that the tendency of their constant employment "is to so deplete the system as to make it a ready prey of disease; to arrest the peristaltic motion of the stomach; to render the emulsification of all food imperfect and ineffectual; to drain the whole intestinal canal of vital fluid; to compel the most dangerous form of hemorrhoids; to induce fatal anemic conditions; in short, to destroy human life;" and we are equally satisfied that it makes small difference whether the excretory stimulant employed accomplishes its fell purpose chemically or mechanically, by blistering, or scratching—the injurious tendencies being identical. We here emphasise what we uttered last October.

But, while calling us stupid and cruelly malicious—pleasant phrases, surely—because we see fit to utter a few gentle truths about the "noble" Graham, our Battle Creek friend kindly admits much that we claim, in the following words:

"We have nothing to say against preparations of the grains from which the outer hull has been removed. In some cases such preparations are greatly preferable to those which contain the whole bran."

Now, if we could only convince this writer that the object of eating is to take food—nutriment, not trash—into the system, we should be charmed. It may be difficult, but we have hopes. If he will only exclude all bran from his own and his patients' food for a few months, we are positive that he will not go back to the sand-paper system any more. But let us beg of him not to depend upon sieves to exclude the hulls. If he does, he will lose a good deal of the best food in the grain, the gluten, which adheres very tenaciously to the bran, and is sifted out with it. He must peel off the hull before the grain is pulverized, just as the Health Food Company is doing to the extent of 250 bushels a day. Then he will have a food fit for the gods, or, what is better, fit for godly men and women. Then will our reformer be so far reformed that he will not say foolish or flippant or ugly things of older and wiser and more industrious investigators. Then will his ink shed no gall of bitterness over honest toilers who find his conclusions erroneous and his assumptions unsustained by science. Then shall the waters of Battle Creek run placidly to meet the sea, and the teeming millions upon its banks shall learn unholy and misdirected war no more.

